# CXC‐ receptor 2 promotes extracellular matrix production and attenuates migration in peripapillary human scleral fibroblasts under mechanical strain

**DOI:** 10.1111/jcmm.17609

**Published:** 2022-11-08

**Authors:** Chen Qiu, Chuandong Wang, Xinghuai Sun, Jianjiang Xu, Jihong Wu, Rong Zhang, Gang Li, Kang Xue, Xiaoling Zhang, Shaohong Qian

**Affiliations:** ^1^ Department of Ophthalmology and Vision Science, Eye and Ear, Nose, Throat Hospital, Shanghai Medical College Fudan University Shanghai China; ^2^ NHC Key Laboratory of Myopia Fudan University Shanghai China; ^3^ Laboratory of Myopia Chinese Academy of Medical Sciences Shanghai China; ^4^ Shanghai Key Laboratory of Visual Impairment and Restoration Fudan University Shanghai China; ^5^ Department of Orthopedic Surgery Xin Hua Hospital Affiliated to Shanghai Jiaotong University School of Medicine Shanghai China; ^6^ State Key Laboratory of Medical Neurobiology, Institutes of Brain Science Fudan University Shanghai China

**Keywords:** CXC‐receptor 2, fibroblast, glaucoma, mechanical strain, sclera

## Abstract

As the main loading‐bearing tissue of eye, sclera exerts an important role in the pathophysiology of glaucoma. Intraocular pressure (IOP) generates mechanical strain on sclera. Recent studies have demonstrated that sclera, especially the peripapillary sclera, undergoes complicated remodelling under the mechanical strain. However, the mechanisms of the hypertensive scleral remodelling in human eyes remained uncertain. In this study, peripapillary human scleral fibroblasts (ppHSFs) were applied cyclic mechanical strain by Flexcell‐5000™ tension system. We found that CXC‐ ligands and CXCR2 were differentially expressed after strain. Increased cell proliferation and inhibited cell motility were observed when CXCR2 was upregulated under the strain, whereas cell proliferation and motility did not have a significant change when CXCR2 was knocked down. CXCR2 could facilitate cell proliferation ability, modulate the mRNA and protein expressions of type I collagen and matrix metalloproteinase 2 via JAK1/2‐STAT3 signalling pathway. In addition, CXCR2 might inhibit cell migration via FAK/MLC_2_ pathway. Taken together, CXCR2 regulated protein production and affected cell behaviours of ppHSFs. It might be a potential therapeutic target for the hypertensive scleral remodelling.

## 1 INTRODUCTION

Intraocular pressure (IOP) is the primary risk factor for the pathophysiology of glaucoma, affecting not only the apoptosis of retinal ganglion cells (RGCs), but also the biomechanical behaviours of the optic nerve head (ONH). The lamina cribrosa and peripapillary sclera (PPS) mainly constitute the ONH complex, which may have great influence on the development and progression of glaucoma.^1^, ^2^, ^3^, ^4^ Ocular‐hypertension‐induced alterations in the lamina cribrosa have been numerously documented previously.^5^, ^6^, ^7^ Recent findings have generated increased interest in the biomechanical properties of the hypertensive sclera and exploring its relationship with glaucoma.^8^, ^9^, ^10^ Sclera, especially the PPS, may even exert a more important role than the lamina cribrosa in the pathophysiology of glaucoma.^4^, ^8^, ^9^ Sclera undergoes dynamic alterations with the fluctuation of IOP, including an increased scleral stiffness,^11^, ^12^ complex components changes^13^, ^14^, ^15^ and therefore, how it responds to IOP. Some scholars have proposed that sclera‐based therapy might become a promising approach for RGCs' reservation by intervening scleral remodelling. However, studies with regard to remodelling of the hypertensive sclera, especially in human beings, are far from thorough investigated.

Extracellular matrix (ECM) is important for sclera to maintain the tension generated by IOP. The elevated IOP may increase fibrous components (such as collagens) and decrease non‐fibrous components in mice sclera.^15^ We also confirmed increased expression of type I collagen in chronic ocular hypertension model of rats in our preliminary work.^14^ Hence, we may speculate that the hypertensive scleral alterations probably indicate ECM synthesis rather than degradation, at least in the early stage. Responses of the cells resided in the sclera are the key points. Studies regarding scleral remodelling in human beings were limited to postmortem histology and biomechanical tests. Therefore, mechanistic insight into the driving force of scleral ECM remodelling demands detailed investigation on human scleral fibroblasts (HSFs), especially the peripapillary HSFs (ppHSFs), under the mechanical strain in vitro.

To investigate the ECM remodelling of the hypertensive PPS, it is necessary to establish a proper strain model for ppHSFs in vitro, which could mimic the in vivo ocular hypertension. The Flexcell‐5000™ tension system has been described previously.^16^, ^17^, ^18^, ^19^, ^20^ It is a computer‐controlled vacuum unit conventionally used to apply mechanical forces on different kinds of cells.^18^, ^19^, ^20^ Under a proper strain, the mRNA and protein expressions of the ppHSFs should coincide with the scleral ECM alterations detected in vivo. The mechanical strain also possesses the ability to alter cell behaviours. Therefore, we aimed to (1) explore a proper biaxial mechanical strain for ppHSFs in vitro; (2) screen for the mechanically stimulated genes of ppHSFs under the strain; (3) investigate the effect of CXCR2 on cell behaviours and ECM production under the strain.

In the current study, we employed a precise measurement of the entire transcriptome by high‐throughput sequence. Our results showed that the CXC‐motif chemokine ligands and receptors were the most changed ones and CXCR2 was validated as the most differentially expressed CXC‐ receptor. The CXC‐ligands/receptor axis has been implicated as proinflammatory process and exerts crucial influence on cell proliferation, migration and angiogenesis.^21^, ^22^, ^23^ In our study, we further explored the potential role of CXCR2 on ECM remodelling and cell behaviours under the mechanical strain, including cell proliferation, apoptosis and migration ability.

## 2 MATERIALS AND METHODS

The study was approved by the Institutional Review Board and Ethics Committee of Eye and Ear, Nose, Throat Hospital of Fudan University. Consent to use for research purpose was made from human donors or the family members before conducting the experiment.

### 2.1 Cell cultivation and treatment

Cell culture and identification have been described previously.^16^ In brief, ppHSFs were isolated from the PPS of postmortem human eyes (2 mm scleral band from the ONH^17^) according to a collagenase digestive protocol (Serva). The primary ppHSFs were grown with Dulbecco's modified Eagle's medium (DMEM) containing 20% fetal bovine serum (FBS) and 1% penicillin–streptomycin (Hyclone), in an atmosphere of 5% CO_2_‐95% air in a humidified incubator. Cells after passage 1 were grown in medium containing 15% FBS. The cells used for experiments were between passage 4 and 8. In selective experiments with inhibitors, ppHSFs were incubated with JAK1/2 inhibitor Ruxolitinib (20 μm) for 4 h or FAK inhibitor PF‐573228 (10 μm) for 24 h (both Selleck) before subsequent processing.

### 2.2 Mechanical strain

To explore a proper strain parameter that represented the in vivo elevated IOPs, we attempted to apply multiple mechanical strains by the Flexcell FX‐5000™ tension system (Flexcell International Corporation). The ppHSFs were seeded into the six‐well collagen‐I coated Bioflex plates (Flexcell International Corporation), with 2.5 × 10^5^ cells per well. After the cells were attached to the plates under 24‐h culturing with conventional serum‐containing DMEM, the media were changed with serum‐free DMEM for 24 h, then DMEM containing 1% FBS before the biaxial strain. In the present study, we applied 0, 5% 0.5 Hz, 10% 0.5 Hz, 20% 0.5 Hz strain for 8 and 24 h, respectively.

### 2.3 Reverse transcription–quantitative polymerase chain reaction (RT‐qPCR)

Samples were dissolved in TRIzol reagent (Invitrogen) to extract total RNA. cDNA synthesis was conducted by reversely transcribing 1 ug total RNA into cDNA according to the PrimeScript RT reagent kit (Takara). RT‐qPCR was performed on ABI ViiA7 Real‐Time PCR system (Thermo Lifetech) using SYBR Premix Ex Taq™ (Takara) according to the manufacturer's protocol. PCR parameters used were as follows: 95°C for 30 s, then 50 cycles of 95°C for 5 s and 60°C for 30 s. The forward and reverse primers were described in Table 1. Data were analysed using the comparison Ct (2^−ΔΔCt^) method, and the relative mRNA expressions were normalized to GAPDH.

**TABLE 1:** Primer for polymerase chain reaction.

	Gene Sequence (5′–3′)	
GAPDH	Forward	ACAACTTTGGTATCGTGGAAGG
	Reverse	GCCATCACGCCACAGTTTC
COL1	Forward	GGACACAGAGGTTTCAGTGGT
	Reverse	AGTAGCACCATCATTTCCACGA
COL2	Forward	GCAGCAAGAGCAAGGAGAAG
	Reverse	GCGTAGGAAGGTCATCTGGA
COL3	Forward	TTGAAGGAGGATGTTCCCATCT
	Reverse	ACAGACACATATTTGGCATGGTT
COL4	Forward	GTTGGGCCTCCAGGATTTAC
	Reverse	CTTGGTCACCCTTGTCACCT
MMP2	Forward	TATGGCTTCTGCCCTGAGAC
	Reverse	CACACCACATCTTTCCGTCA
MMP9	Forward	AGTCCACCCTTGTGCTCTTC
	Reverse	ACTCTCCACGCATCTCTGC
ELN	Forward	TCCAGGTGTAGGTGGAGCTT
	Reverse	GTGTAGGGCAGTCCATAGCC
alpha‐SMA	Forward	CCGGGACTAAGACGGGAATC
	Reverse	CACCATCACCCCCTGATGTC
DCN	Forward	ATGCAGCTAGCCTGAAAGGA
	Reverse	GTCCAAGTGAAGCTCCCTCA
ACAN	Forward	GTATGTGAGGAGGGCTGGAA
	Reverse	ATGCTGCTCAGGTGTGACTG
FN1	Forward	CAAACTCCGTCACCCTCAGT
	Reverse	GGTGCCAGTGGTTTCTTGTT
BGN	Forward	TCTCAGAGGCCAAGCTGACT
	Reverse	AGCTTGGAGTAGCGAAGCAG

Abbreviation: ACAN, aggrecan; BGN, biglycan; COL1–COL4, type I–IV collagen; DCN, decorin; ELN, elastin; FN, fibronectin; GAPDH, glyceraldehyde‐3‐phosphate dehydrogenase; MMP, matrix metalloproteinase; SMA, smooth muscle actin.

### 2.4 Western blot

Cells were washed twice with precooled (4°C) PBS and then lysed with radio‐immunoprecipitation assay (RIPA) buffer (Biocolor) containing protease inhibitor phenylmethanesulfonyl fluoride (PMSF; Biosharp; 99:1) for 30 min. Protein concentration was measured using the BCA method (Thermo Fisher Scientific). Each sample that contained 25 μg total proteins was separated with 10% polyacrylamide gel electrophoresis (PAGE) gels and electrotransferred on nitrocellulose membranes (GE Healthcare Life Science). The membranes were blocked with 5% bovine serum albumin (BSA; Sigma‐Aldrich Corp.) for 1 h at room temperature and then incubated overnight at 4°C with the primary antibodies against collagen I, MMP2 (1:1000; Novus), CXCR2, phospho‐STAT3, STAT3, phospho‐FAK, FAK, α‐SMA (1:1000; Abcam), phospho‐AKT, AKT, phospho‐ERK1/2, ERK1/2, phospho‐MLC_2_, MLC_2_ (1:1000, Cell Signalling Technology) and anti‐GAPDH (1:10,000; Bioworld). After washing with PBS for three times, the membranes were incubated with Horseradish peroxidase (HRP)‐conjugated secondary antibody (1:3000; Cell Signaling Technology) for 1 h at room temperature. To visualize the immunoreactivities of the protein bands, Super Signal™ West Femto Substrate Trial Kit (Thermo Fisher) was used for chemiluminescence detection. All the bands were analysed in triplicate with Image J software (National Institutes of Health). GAPDH was set as the endogenous control.

### 2.5 RNA‐sequence (RNA‐seq) and the bioinformatics analysis

Three pairs of cell samples were collected from the mechanical stimulated ppHSFs (10% 0.5 Hz strain for 8 h) or the unstimulated normal control. Total RNA was extracted by TRIzol reagent (Invitrogen). After samples' quality certification, RNA‐seq running was carried out by the Beijing Genomics Institute (BGI) using BGISEQ‐500. The differentially expressed genes (DEGs) with *p*‐value < 0.05, fold change ≥2 or ≤−2 were considered significant. The RNA‐seq data revealed the DEGs and the DEGs were further analysed to identify functionally related gene ontology (GO) categories. Similar GO terms were clusters and assigned an enrichment score, then predict biological significance. Pathway enrichment analysis of DEGs was performed based on Kyoto Encyclopedia of Genes and Genomes (KEGG) database.

### 2.6 Lentivirus transfection

The lentiviral vector containing short‐hair RNA (shRNA) of human CXCR2 and the scramble lentiviral vector were purchased from Genomeditech Co. Ltd. After being seeded in the six‐well plates for 24 h, the ppHSFs were transfected with lentiviral vectors for 24 h, respectively. Then the medium was replenished with DMEM containing 15% FBS and puromycin (4 μg/ml; Genomeditech Co., Ltd.). After 48‐h growth, the cells were resuspended and seeded into the plates for 24‐h culturing before further experiments. After cell harvests, the mRNA and protein expressions of the transfected cells were confirmed by RT‐qPCR and Western blot, respectively.

### 2.7 5′‐ethynyl‐ 2′deoxyuridine (Edu) imaging

Edu imaging was employed to compare cell proliferation abilities between groups (Click‐iT™ Edu Alexa Flour™ 488 imaging kit; Life Technologies). Briefly, the ppHSFs were resuspended and seeded into 96‐well plates. After overnight cell attachments, 10 μm Edu was added into each well and incubated for 4 h, followed by cell fixation and permeation. Then the cocktail was incubated for 30 min to detect the positive cells. Images were taken by fluorescence microscopy (Nikon Eclipse Ti‐S).

### 2.8 Cell cycle by flow cytometry

The experiments for cell cycle of flow cytometry have been conducted following the manufacturer's protocol. In brief, the resuspended ppHSFs were washed twice with ice‐cold PBS and fixed in 75% ice‐cold ethanol overnight at −20°C. After washing with the precooled PBS and then the stain buffer (BD Pharmingen, San Diego, CA, USA), the cells were suspended in propidium iodide (PI)/RNase staining buffer (BD Pharmingen) in the dark for 15 min. Then we conducted the machine test (Gallios; Beckman) immediately. The cell cycle phases were analysed with Modfit software (Verity Software House).

### 2.9 Immunocytochemistry/Immunofluorescence analysis

ppHSFs were cultured in the six‐well Bioflex soft membranes and supplemented with conditional media. The cells were fixed in 4% formaldehyde PBS for 15 min, rinsed twice with PBS, and then the flexible membranes were scissored from the plates for immunofluorescent analysis. The samples were permeabilized with 0.1% Triton X‐100 in PBS for 10 min and blocked with 5% BSA in PBS for 1 h at room temperature. The primary antibody: anti‐phosphorylated STAT3 (1:200) was incubated overnight at 4°C. Subsequently, secondary antibody (Alexa Flour 488‐conjugated goat IgG) was incubated for 1 h at room temperature. Fluorescence imaging was conducted by confocal microscopy (Leica SP8; Leica).

### 2.10 Enzyme‐linked immunosorbent assay (ELISA)

The secretory level of type I collagen was detected by ELISA. Briefly, cell culture medium from different groups was collected immediately. The secretory level of type I collagen was quantitatively determined following the instructions of the ELISA kit (Abcam).

### 2.11 Zymography

The activity of MMP2 was detected by MMP Zymography Assay Kit (Xinfanbio, Shanghai, China). According to the procedure, we collected cell samples from the unstrained and strained groups. Then, 50 μg of the prepared protein lysates from each group was separated by SDS‐PAGE. After gel incubation with solution A and B, respectively, we stained the gels with Coomassie blue for 1 h, then de‐stained them with methanol–acetic acid solution for 2 h. The semitransparent bands were shown, and the quantitative analysis of MMP2 activity was performed by Image J.

### 2.12 Annexin V Alexa Flour™ 488/PI dual staining assay

Cell apoptosis was detected using the Annexin V Alexa Flour™ 488/PI Dual Staining kit (Life Technologies). The ppHSFs were harvested and washed twice with precooled PBS. The cells (1 × 10^6^) were resuspended with 100 μl 1× binding buffer. PI and Annexin V staining were carried out in the dark for 15 min at room temperature. Then, 400 μl 1× binding buffer was added into each sample. Apoptotic cells were analysed with FACScan flow cytometry (Gallios, Beckman) and FlowJo software (BD).

### 2.13 Migration assay

Cell migration ability was determined by the migration assay. After the required treatment of each subgroup, cells (2.5 × 10^5^/well) were transferred into the six‐well plates and cultured with the DMEM containing 10% FBS, then serum‐free DMEM, respectively, for 24 h. Cell migration was observed by the living cells workstation using Leica DMI 6000B microscopy. The pictures were taken every 2 h at exactly the same position and calculated by Photoshop software (Adobe). The pop left corner of the pictures showed the shooting time.

### 2.14 Statistical analysis

The data were presented as mean ± SD, and every experiment was repeated at least three times. Statistically analyses were assessed by student *t* test or one‐way ANOVA using SPSS software (version 19.0; Inc.). *p* value < 0.05 were considered to indicate statistical significance.

## 3 RESULTS

### 3.1 The mRNA and protein expressions of the ppHSFs under multiple mechanical strains

To explore a proper strain parameter, multiple biaxial mechanical strains were applied on the ppHSFs. Fourteen independent cell samples participated in the following experiments: 0, 5% 0.5 Hz, 10% 0.5 Hz, 20% 0.5 Hz strain for 8 h and 24 h, respectively. The mRNA expressions of ECM‐related genes were quantified by qPCR (Figure S1A). Our results showed that these genes underwent dynamic alterations with the effect of different strains. As compared with the unloaded control, the mRNA levels of type I ~ IV collagens, elastin and aggrecan were primary raised first, then decreased, whereas the transcriptional expressions of MMP2, decorin, fibronectin and biglycan were fundamentally decreased when gradually intensifying the strains.

The protein expressions of type I collagen and MMP2 in ppHSFs under multiple strains were consistent with those at the transcriptional level (Figure S1B,C). As the main components in sclera, type I collagen constituted more than 90% of the scleral dry weight.^24^ Type I collagen was 1.59 ± 0.36, 1.62 ± 0.39, 0.58 ± 0.13, 1.12 ± 0.18, 0.90 ± 0.13, 1.40 ± 0.36 fold change under 5% 0.5 Hz, 10% 0.5 Hz, 20% 0.5 Hz strain for 8 and 24 h, respectively. As with myopia, MMP2 may also play a crucial role in modulating the scleral remodelling during ocular hypertension.^14^, ^25^ The relative protein level of MMP2 was 0.45 ± 0.10, 0.60 ± 0.16, 0.83 ± 0.17, 0.91 ± 0.11, 1.17 ± 0.22, 0.99 ± 0.19 fold change, respectively.

According to the documented literatures, the hypertensive sclera revealed increased fibrous components and decreased non‐fibrous components,^15^ accompanying increased stiffness^11^, ^12^ and reduced permeability of sclera.^13^ Our preliminary work has also confirmed the upregulated scleral production of type I collagen and elastin in chronic ocular hypertension model of rats, at least in the early stage.^14^ Taken together, the strain of 10% 0.5 Hz for 8 h may be a proper strain for the ppHSFs, mimicking the hypertensive scleral ECM productions in vivo.

### 3.2 RNA sequence and mRNA expression profiles between the mechanical stimulated and unstimulated ppHSFs

**FIGURE 1:**
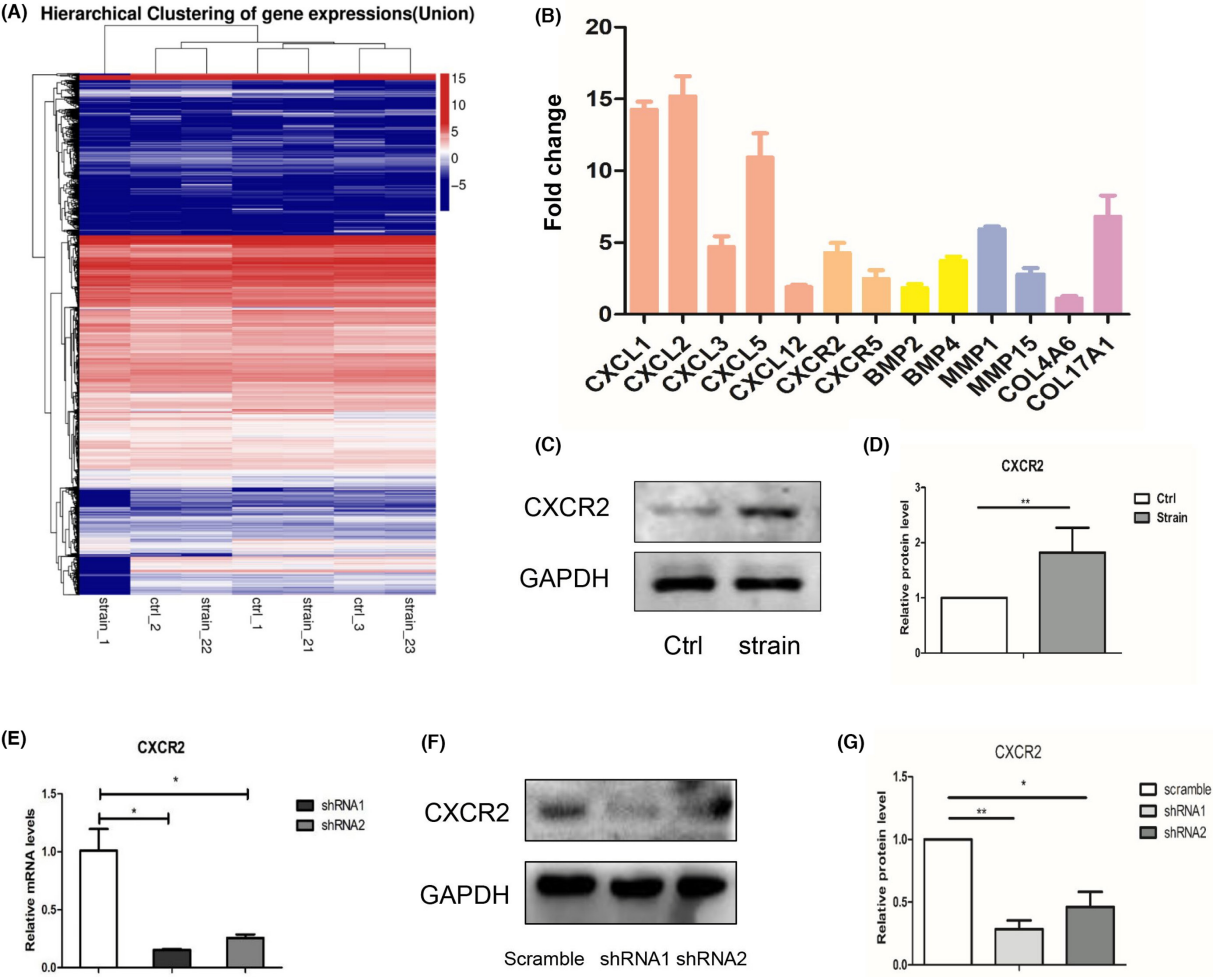
RNA sequence and mRNA expression profiles between the mechanical stimulated and unstimulated ppHSFs. The ppHSFs were subjected to the strain of 10% 0.5 Hz or unloaded for 8 h. (A) High‐throughput mRNA sequence showed the upregulated and downregulated mRNAs between stimulated and unstimulated ppHSFs (*n* = 3). (B) The mRNAs of the most differentially expressed genes were further verified by RT‐qPCR and expressed as the relative expression levels as compared with the unstimulated group (*n* = 11). (C, D) The protein levels and the semi‐quantitative analysis of CXCR2 in ppHSFs under the strain of 0 and 10% 0.5 Hz for 8 h, respectively (*n* = 5). (E–G) The establishment of lentivirus vectors containing the short‐hair RNA of human CXCR2 (*n* = 3). (E) The relative mRNA levels, (F, G) Western blot analysis and the relative protein levels of CXCR2. GAPDH was set as the loading control. Data were expressed as mean ± SD of three replicates. *p < 0.05, **0.001 < *p* < 0.01.

Three independent cell samples were used for the entire transcriptome measurements by high‐throughput sequence. The mRNA expressions were compared between the mechanical stimulated (10% 0.5 Hz for 8 h) and unstimulated ppHSFs. The transcriptome was massively altered after mechanical stretching (Figure 1A). The bioinformatics‐based results revealed that the mechanical stimulation triggered a cluster of proinflammatory cytokines, especially the CXC‐ligands and receptors. We further conducted qPCR with 11 independent samples to verify the sequence data and confirmed that the CXC‐family (CXCL1, CXCL2, CXCL3, CXCL5, CXCL12, CXCR2, CXCR5) was most differentially expressed after the strain (Figure 1B).

The GO and KEGG pathway enrichment analyses are shown in Figure S2. The bioinformatics results predicted a possible gene network under the mechanical strain. The aberrantly stimulated CXCLs combined to the upregulated CXCR2 of ppHSFs, then CXCR2 may transduce the external mechanical signals into the intracellular biological signals, modulate the downstream signalling pathways, thus initiating the inflammatory cascades and altering cell behaviours. Western blot analysis was also performed to visualize the protein expression of CXCR2 under the strain (Figure 1C‐D). Therefore, CXCR2 may be an important target for ppHSFs under the stimulation. The demographic features of donors for mRNA sequence and qPCR validation are listed in Table 2.

**TABLE 2:** Demographic information of human donors.

No	Age, year	Race	Gender	No	Age, year	Race	Gender
1	45	Han	Female	8	68	Han	Female
2	61	Han	Male	9	42	Han	Male
3	67	Han	Male	10	40	Han	Male
4	69	Han	Male	11	78	Han	Male
5	62	Han	Female	12	52	Han	Female
6	86	Han	Male	13	82	Han	Male
7	67	Han	Male	14	57	Han	Male

To further investigate the function of CXCR2 in ppHSFs, the lentivirus vectors containing shRNA of human CXCR2 gene were established. shRNA1 was selected for subsequent experiments as it was more effective in knocking down CXCR2 (Figure 1E–G).

### 3.3 The mechanically stimulated CXCR2 prompted cell proliferation of ppHSFs under the mechanical strain

**FIGURE 2:**
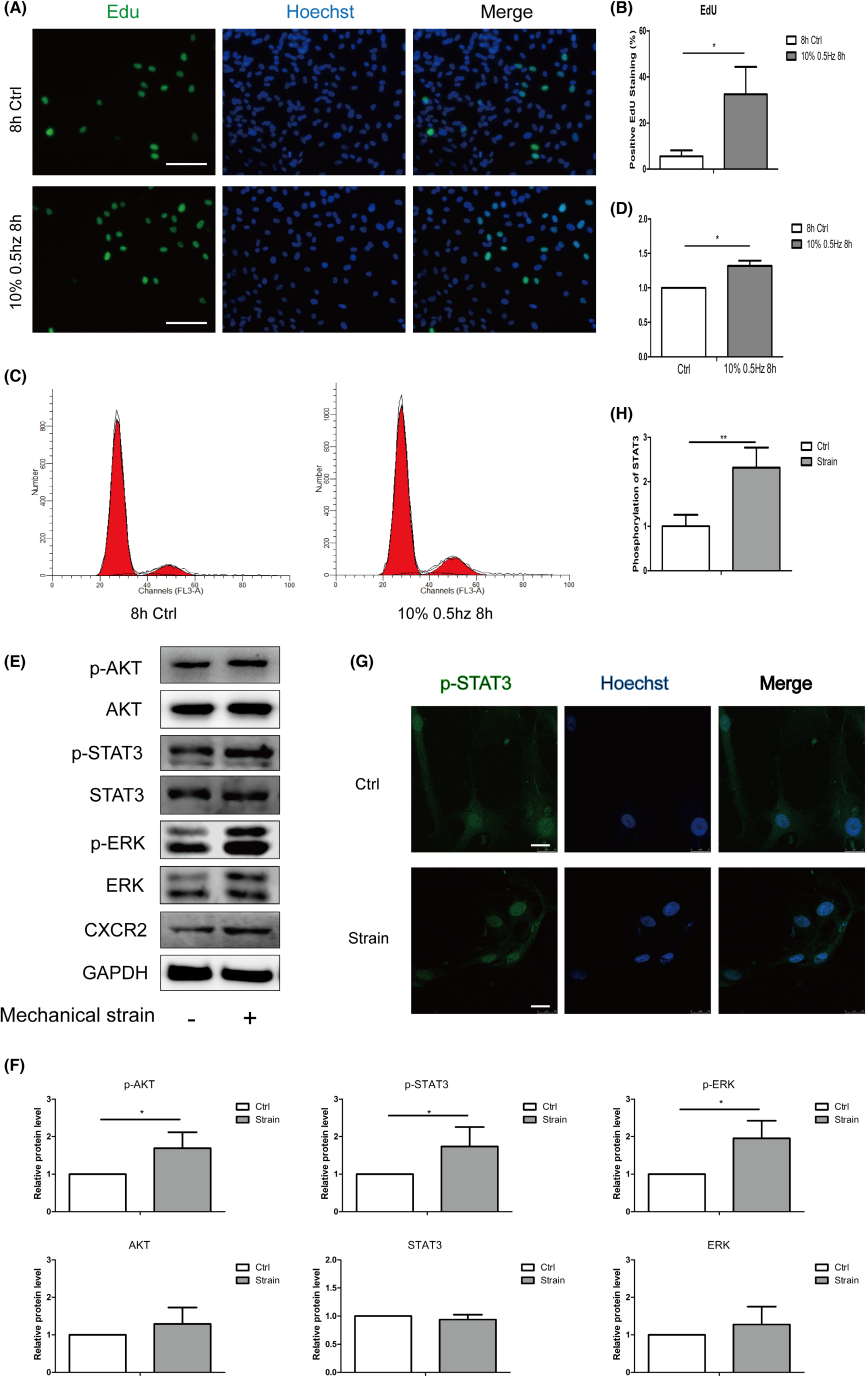
Mechanically stimulated upregulation of CXCR2 prompted cell proliferation of ppHSFs. The ppHSFs were stretched under the strain of 10% 0.5 Hz for 8 h or set as the unstretched control. CXCR2 was upregulated after the strain. (A) Edu staining of the unstimulated and stimulated ppHSFs, respectively. *Scale bar*: 100 μm. (B) Quantification of the proportion of positive Edu staining (*n* = 3). (C, D) Cell cycle analysis conducted by flow cytometry and the quantitative data (*n* = 5). (E) Western blot and (F) the semiquantitative analysis showed that several signalling pathways may be involved in the ppHSFs when CXCR2 was increased after strain (*n* = 5). (G) Confocal immunofluorescent images of the phosphorylated STAT3 (green) and nuclei (blue) of the ppHSFs. (H) Semi‐quantification of the phosphorylated STAT3 in nuclei (*n* = 5). *Scale bar*: 25 μm. GAPDH was set as the loading control. Data were expressed as mean ± SD of three replicates. *p < 0.05, **0.001 < *p* < 0.01.

**FIGURE 3:**
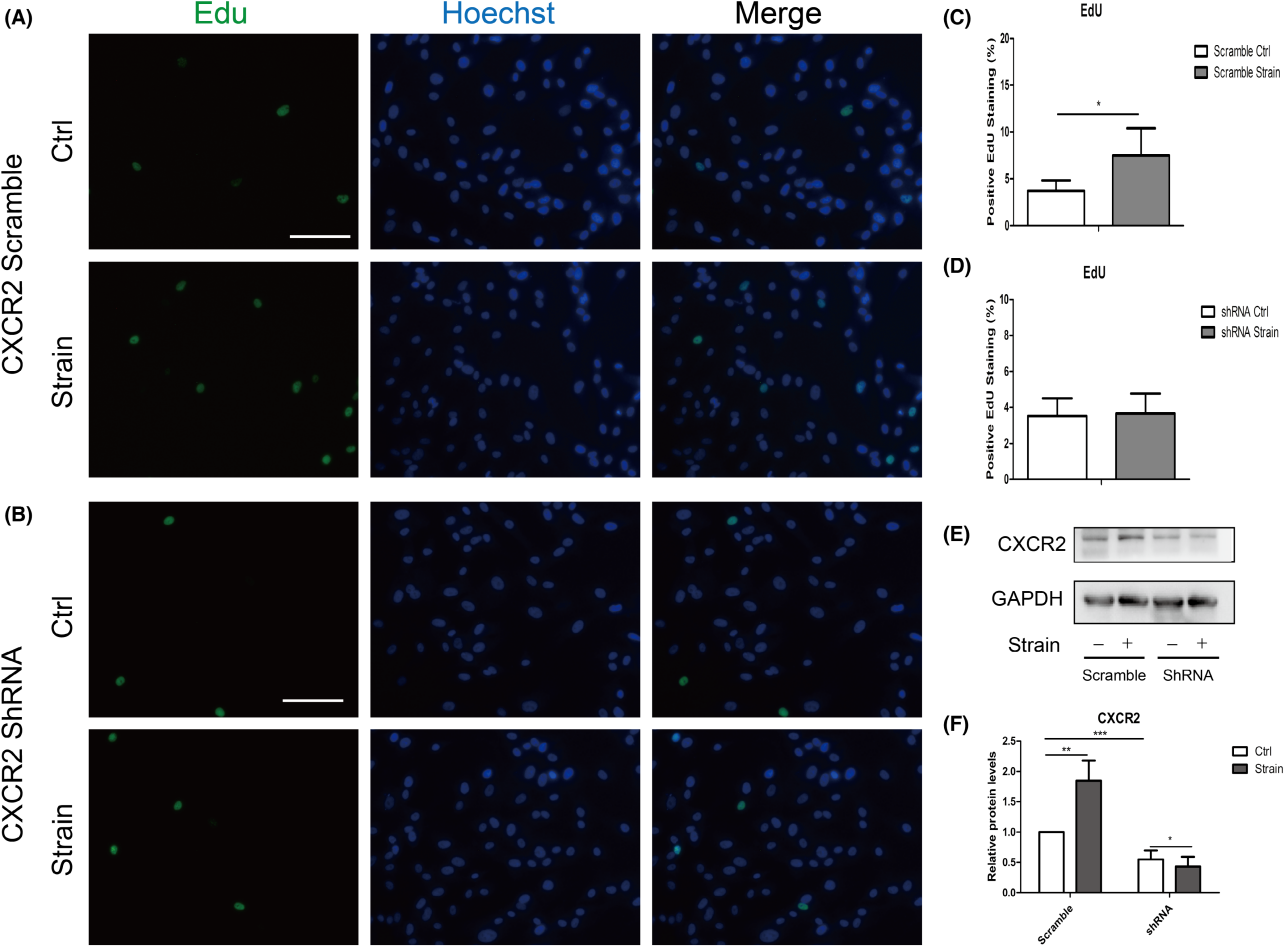
Knockdown of CXCR2 attenuated cell proliferation of ppHSFs under the mechanical strain. Lentivirus vectors were pretreated before the strain. The ppHSFs from the scramble and CXCR2 shRNA group were subjected to the strain of 10% 0.5 Hz or unloaded for 8 h, respectively. (A, B) Edu imaging of the ppHSFs in the scramble and shRNA group, with or without strain, respectively (*n* = 4). (C, D) Edu imaging quantification of the ppHSFs in each group, with or without strain (*n* = 4). (E, F) Western blot band and semi‐quantification of CXCR2 as compared with GAPDH (*n* = 4). Data were expressed as mean ± SD of three replicates. *p < 0.05, **0.001 < *p* < 0.01, and ***p < 0.001. *Scale bar*: 100 μm.

Under the strain of 10% 0.5 Hz for 8 h, the expression of CXCR2 was upregulated in ppHSFs. However, the effect of CXCR2 on ppHSFs has not been well explored. Edu imaging and cell cycle analysis of flow cytometry were performed to detect cell proliferation ability. When compared with the unstimulated normal control, the proportion of positive Edu cells (green) was increased in the stimulated group, indicating prompted cell proliferation (Figure 2A,B). Cell cycle analysis of flow cytometry also showed a significant increase in G2 and S phase in ppHSFs (Figure 2C,D). Our data also revealed that when CXCR2 was upregulated, the phosphorylation of AKT, STAT3 and ERK1/2 was increased, which might explain the stimulated cell proliferation (Figure 2E,F). The immunofluorescent imaging showed nuclei translocation of the phosphorylated STAT3 under the same circumstances (Figure 2G). When CXCR2 expression was knocked down by shRNA, Edu imaging did not have a significant change between the two groups (Figure 3). Therefore, CXCR2 might promote cell proliferation of ppHSFs under the mechanical strain.

### 3.4 The mechanically stimulated CXCR2 increased scleral ECM production in ppHSFs under the mechanical strain via Jak1/2‐STAT3 signalling pathway

**FIGURE 4:**
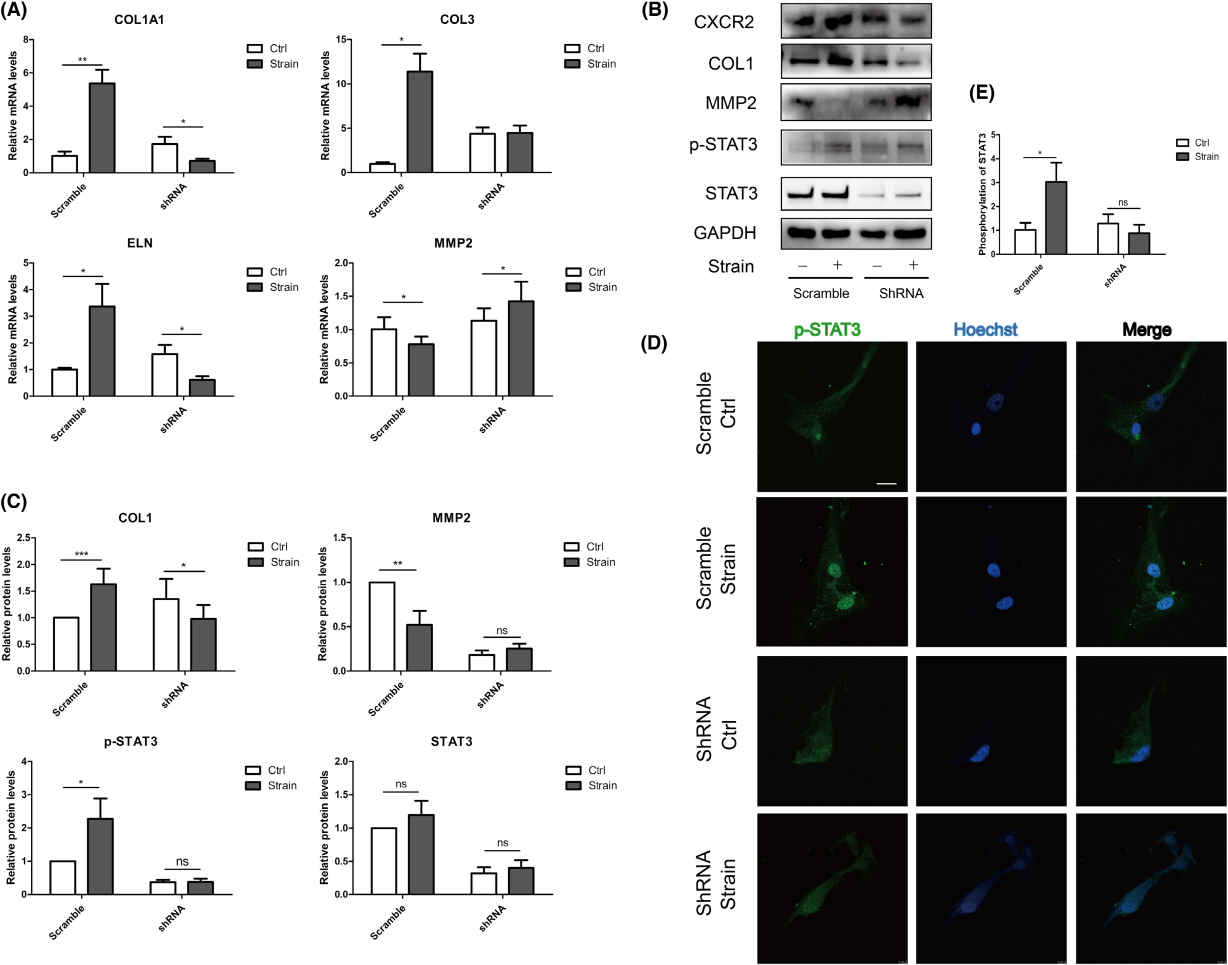
The mechanically stimulated CXCR2 increased scleral ECM production in ppHSFs under the mechanical strain. CXCR2 was knocked down by the lentivirus vectors containing the shRNA of human CXCR2. ppHSFs from the scramble and shRNA group were mechanically stimulated (10% 0.5 Hz strain for 8 h) or unstimulated, respectively. (A) qPCR showed the mRNA expressions of collagen I, collagen III, elastin and MMP2 (*n* = 5). (B) Western blot and (C) the quantification analysis of the protein expressions in ppHSFs (*n* = 5). GAPDH was set as the loading control. (D) Confocal immunofluorescent images of the phosphorylated STAT3 (green) and nuclei (blue) of the ppHSFs in scramble and shRNA group, respectively. (E) Semi‐quantification of the phosphorylated STAT3 in nuclei (*n* = 6). *Scale bar*: 25 μm. Data were expressed as mean ± SD of three replicates. *p < 0.05, **0.001 < *p* < 0.01, and ***p < 0.001; ns, no significant.

To explore the effect of CXCR2 on the mRNA and protein productions of scleral ECM, qPCR and Western blots were conducted. The results revealed that strain‐stimulated CXCR2 upregulation increased the mRNA and protein productions of type I collagen and reduced the production of MMP2 in the scramble group. When CXCR2 expression was inhibited, the mRNA and protein productions of type I collagen and MMP2 were reversed after the stimulation (Figure 4A–C). CXCR2 could also modulate the secretory level of type I collagen (Figure S3) and the activity of MMP2 (Figure S4A,B). The translocation of phosphorylated STAT3 under the strain was also attenuated when CXCR2 was knocked down (Figure 4D,E).

**FIGURE 5:**
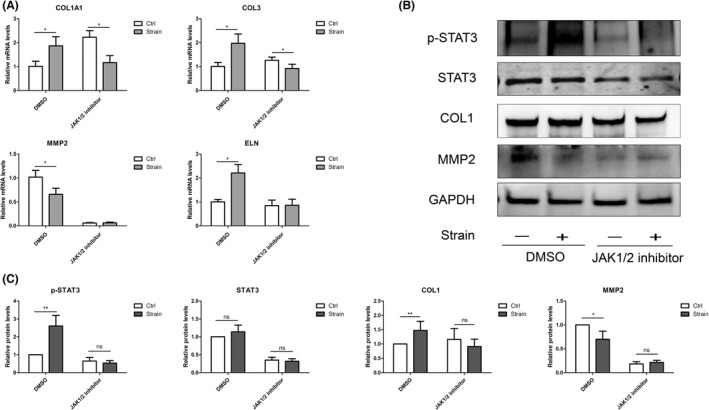
Inhibition of JAK1/2 attenuated the scleral ECM production in ppHSFs under the mechanical strain. The ppHSFs were pretreated with Ruxolitinib (20 μm) or DMSO (control) for 4 h before the strain. The cells were then applied with 10% 0.5 Hz strain for 8 h or used as unstimulated control. (A) The mRNA expressions of collagen I, collagen III, elastin and MMP2 (*n* = 5). (B, C) Western blot bands and the quantitative results of the protein levels in ppHSFs (*n* = 4). *p < 0.05, **0.001 < *p* < 0.01; ns, no significant.

Increased type I collagen, reduced MMP2 and activated phosphorylation of STAT3 under the strain could also been abrogated by applying JAK1/2 inhibitor Ruxolitinib (Figure 5). Thus, we humbly speculated that the mechanically stimulated CXCR2 might modulate scleral ECM production in ppHSFs via JAK1/2‐STAT3 signalling pathway.

### 3.5 The effect of CXCR2 on cell apoptosis of ppHSFs

Flow cytometry of annexin V/PI dual staining was used to analyse cell apoptosis. The apoptosis of ppHSFs was slightly reduced under the strain, although without statistical significance (*p* = 0.875). In the shRNA interfering group, the expression of CXCR2 was inhibited and apoptotic cells were significantly reduced after strain (*p* = 0.042; Figure S5). The results implied that CXCR2 inhibition might reduce the apoptosis of ppHSFs.

### 3.6 The mechanically stimulated CXCR2 attenuated cell migration of ppHSFs under the mechanical strain via FAK/MLC_2_ pathway

**FIGURE 6:**
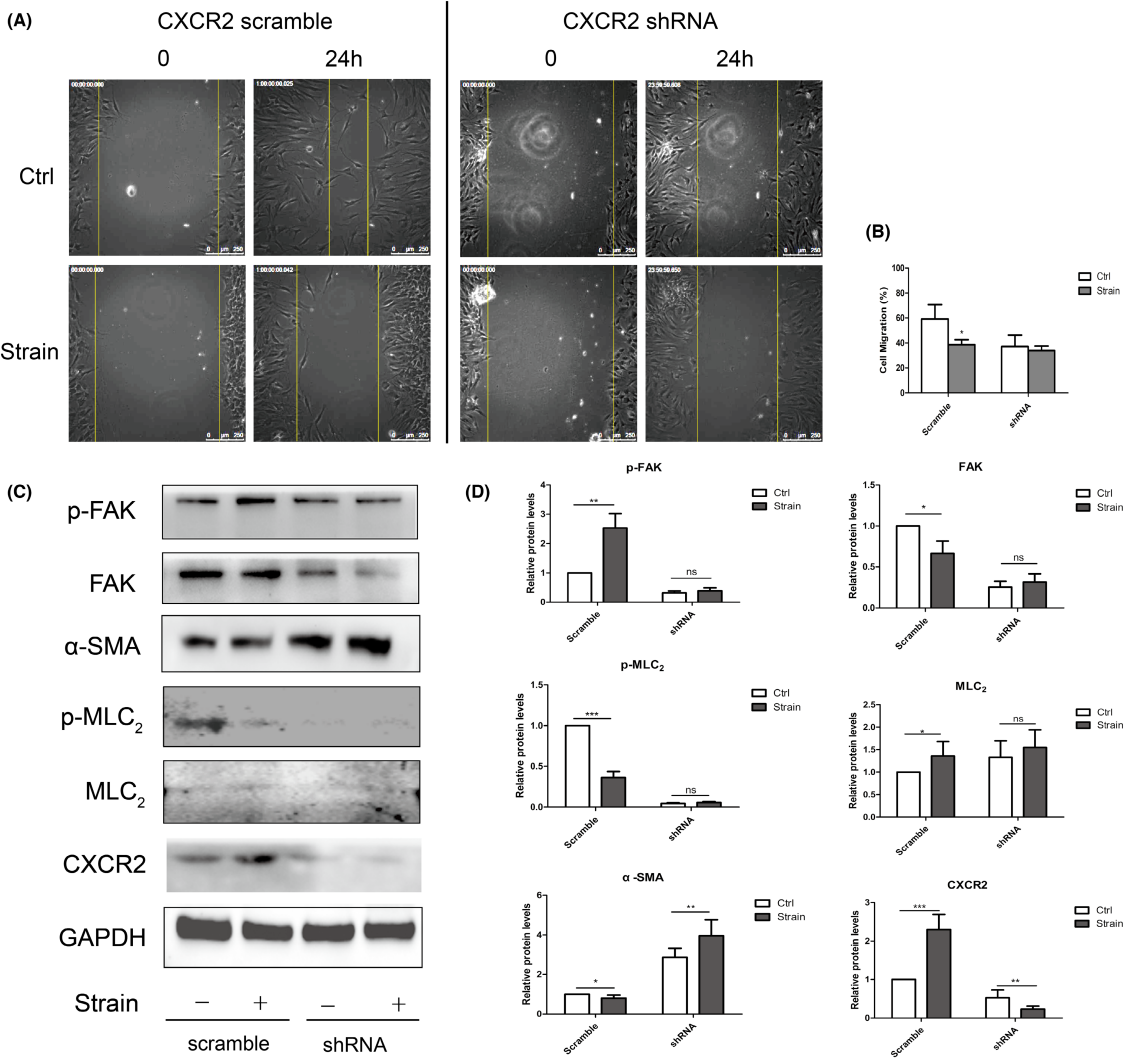
The mechanically stimulated CXCR2 retarded cell migration in ppHSFs under the mechanical strain. CXCR2 was knocked down by the lentivirus vectors containing the shRNA of human CXCR2. The ppHSFs from the scramble and shRNA group were stimulated with 10% 0.5 Hz strain for 8 h or unstimulated, respectively. (A, B) Comparison and quantification of cell migration between the scramble and shRNA group in ppHSFs, with or without strain (*n* = 5). Cell migration was observed by the living cells workstation using Leica DMI 6000B microscopy. The pictures were taken every 2 h at exactly the same position, with shooting time shown on the top left corner. *Scale bar*: 250 μm. (C) Western blot showed the protein expressions of phosphorylated FAK, FAK, α‐SMA, phosphorylated MLC_2_, MLC_2_ and CXCR2_._ (D) Semi‐quantitative analysis of the protein levels (*n* = 5). GAPDH was used as the loading control. Data were expressed as mean ± SD of three replicates. *p < 0.05, **0.001 < *p* < 0.01, and ***p < 0.001; ns, no significant.

For cell mobility, migration assay was performed. In the scramble group, ppHSFs migrated 38.65% and 59.16% of the total distance 24 h after the scratch, respectively, with or without strain (*p* = 0.036). In the shRNA group, the cells migrated 34.06% and 37.12% of the total distance, respectively, with or without strain (*p* = 0.547; Figure 6A,B). Under the mechanical strain, the upregulation of CXCR2 increased the phosphorylated FAK but inhibited the phosphorylated MLC_2_, hence reduced the migration ability of ppHSFs. However, the phosphorylation of FAK and MLC_2_ did not have a significant change after strain when CXCR2 was knocked down (Figure 6C,D). Therefore, CXCR2 may affect the migration ability of ppHSFs under the mechanical strain.

**FIGURE 7:**
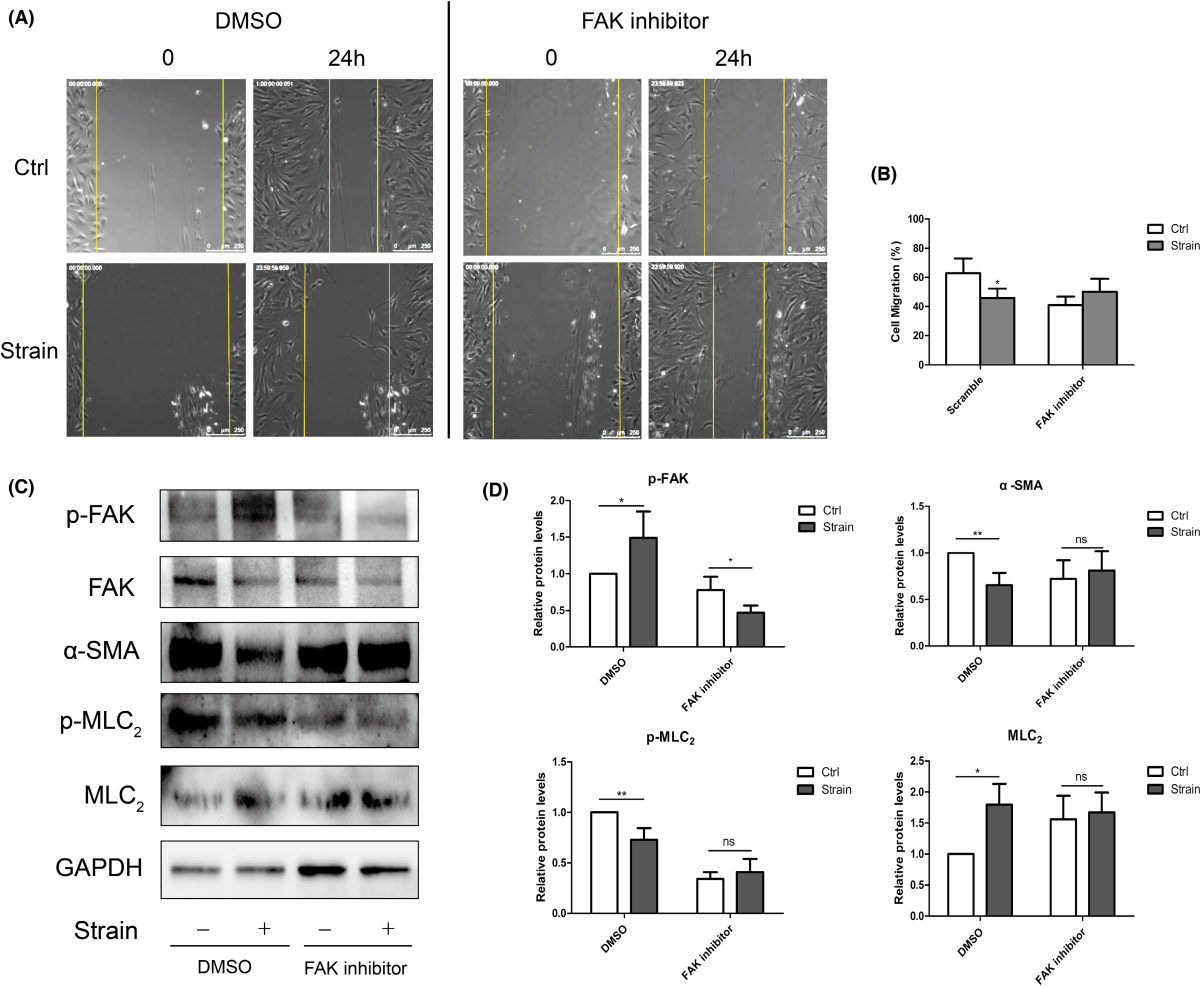
Inhibition of FAK resumed cell migration ability of ppHSFs under the mechanical strain. The ppHSFs were pretreated with PF‐573228 (10 μm) or DMSO (control) for 24 h before the strain. Strain parameter: 10% 0.5 Hz strain for 8 h. (A) FAK inhibitor attenuated the decreased cell migration of ppHSFs after the strain. Cell migration was observed by the living cells workstation using Leica DMI 6000B microscopy. The pictures were taken every 2 h at exactly the same position, with shooting time shown on the top left corner. *Scale bar*: 250 μm. (B) Quantification of the proportion of migrated distance to total distance (*n* = 4). (C) Western blot and (D) the quantification analysis of the protein levels in DMSO and FAK inhibitor group, respectively, with or without strain (*n* = 4). Relative protein levels were determined by normalizing to GAPDH. Data were expressed as mean ± SD of three replicates. *p < 0.05, **0.001 < *p* < 0.01; ns, no significant.

Further, FAK inhibitor and DMSO were applied. In the control (DMSO) group, cell migration was slow down under the strain. Consistently, the phosphorylation of FAK upregulated and the phosphorylation of MLC_2_ downregulated, consistent with the results in CXCR2 scramble group (Figure 7). However, cell migration and the phosphorylation of MLC_2_ were resumed after applying FAK inhibitor (Figure 7). Taken together, CXCR2 may inhibited the migration of ppHSFs under the strain through FAK/MLC_2_ signalling pathway.

## 4 DISCUSSION

Given that PPS plays an important role in sustaining the biomechanical structure of the ONH, it is essential to explore the underlying molecular mechanisms of the hypertensive scleral remodelling. In this study, we found that the mRNA and protein expressions of ppHSFs varied under multiple strains in vitro, based on which we selected a proper strain parameter that simulated the in vivo hypertensive scleral ECM remodelling. Under the strain of 10% 0.5 Hz for 8 h, CXCR2 was significantly upregulated and validated as a mechanosensitive gene of ppHSFs. The mechanically stimulated CXCR2 could increase cell proliferation, promote ECM components' production, but inhibit cell migration (Figure S6). Interfering the expression of CXCR2, the proliferation and motility of ppHSFs did not have a significant change under the strain. These results suggested that CXCR2 might be a mechanical biomarker and a potential therapeutic target for the remodelling of hypertensive sclera.

The ligands and receptors of CXC‐ are key regulators in carcinogenesis, metastasis and inflammation. CXCR2, as a G protein receptor, may exist on the membranes of a variety of cells, such as neutrophils,^26^, ^27^ fibroblasts^22^ and endothelial cells.^28^ The CXC‐ligands and CXCR2 expression could recruit neutrophils and granulocytes to the inflammatory sites.^27^, ^29^ Overexpression of CXCR2 could promote cell proliferation, invasion and migration.^21^, ^22^, ^23^ Additionally, depletion of CXCR2 may retard wound healing,^30^ reduce collagen deposition^31^ and inflammation infiltration.^32^ CXCR2 may also activate a variety of downstream proteins, such as NF‐κB,^33^, ^34^ bcl‐xL,^35^ β‐catenin,^36^ Src.^37^ Zhou et al.^33^ found that CXCR2 might induce neuralgia by activating NF‐κB after exposure to the vincristine. CXCR2 could also stimulate the expression of STAT in diabetic animal models, contributing to activation of the glomerular monocytes and migration of the macrophages.^38^ By upregulating β‐catenin signalling pathway, CXCR2 increased migration, invasion and epithelial–mesenchymal transition of the papillary thyroid carcinoma cells.^36^

The crucial role of CXCR2 was also detected in eye diseases, such as keratitis,^34^ proliferative vitreoretinopathy^39^ and so on. However, the effect of CXCR2 on sclera remains unclear. Our RNA‐seq results showed that the levels of CXCLs and CXCR2 in ppHSFs were the most differentially expressed after the strain. Although the strain stimulated mild upregulation of CXCR2, the alteration was effective enough to initiate a series of complicated molecular networks. CXCR2 could increase cell viability of the ppHSFs under the strain, with more production of ECM proteins by activating the phosphorylation of STAT3. However, the phenomena were blocked by inhibiting JAK1/2‐STAT3 signalling pathway, a canonical signalling pathway that regulates cell proliferation and inflammation.^40^ Under the mechanical strain, activation of JAK recruited and phosphorylated STAT3. The phosphorylated STAT3 entered into the nuclei and altered gene transcription. Taken together, the upregulated CXCR2 may promote cell proliferation and stimulate ECM production of ppHSFs via JAK1/2‐STAT3 signalling pathway under the mechanical strain.

To furtherly understand how CXCR2 retarded the migration of ppHSFs, much emphasis is given on FAK.^41^ Stimulation of integrins, growth factors and G protein‐coupled receptors could induce FAK phosphorylation. The CXC‐ligands could also activate and phosphorylate FAK by binding with CXCR2.^42^, ^43^ In the present study, the RNA‐seq analysis showed that CXCR2/CXCLs were the most differentially expressed. And the knockdown of CXCR2 validated the role of CXCR2 in regulating FAK. As a tyrosine kinase, FAK has been reported to modulate cell adhesion and migration by changing cell adhesion and cytoskeletons. By repeated circles of attachment and unattachment with cytoskeletons, especially the actins and microtubules, cell migrated. MLC is required for actin‐myosin contractility. Phosphorylated (contractile) state of MLC_2_ controls the activity of myosin II, a key component of focal adhesion complex and stress fibre formation.^44^, ^45^, ^46^ Inhibition of myosin II could alleviate cell motility.^45^ Some scholars have reported that FAK stimulation augmented the expression of MLC.^41^ However, we found that CXCR2‐activated FAK downregulated MLC_2_ activity and repressed cell motility of ppHSFs under mechanical strain in the present study. After applying FAK inhibitor, the decreased cell migration was attenuated after the strain. The opposing effects of CXCR2‐mediated FAK‐MLC_2_ signalling pathway on cell migration may depend on the cues delivered to the cells. FAK had six potential tyrosine phosphorylation sites. Skewing of the phosphorylation sites of FAK may perturbate cell migration and adhesion. FAK over‐phosphorylation may result in increased adhesion and inhibited cell motility in rat basophilic leukaemia cells,^42^ which was in line with our findings. We speculated that discrepancies in cell migration might originate from the phosphorylation sites of FAK. It could also be explained that the stimulated ppHSFs might prevent FAK undergoing dephosphorylation, leading to imbalance of attachment between FAK and actins. Another explanation may be the distinctive characteristics of HSFs in PPS, especially after long‐term exposure to mechanical stimulation in vivo. Our previous work has shown that cells in the periphery sclera were distinct from those in the PPS, and they reacted differently even under the same mechanical stimulation, including the expression of α‐SMA.^16^ Further investigations would be conducted to full elucidate the CXCR2‐FAK‐MLC signalling pathway. Generally, the reduced migration was a well‐coordinated process. Increased scleral ECM production and retarded cell motility may together contribute to the remodelling of the hypertensive sclera.

Taken together, our study showed that the strain of 10% 0.5 Hz for 8 h might be a proper condition for ppHSFs to reproduce the in vivo hypertensive scleral remodelling. Under the strain, ppHSFs may undergo a complicated inflammatory process, at least in the early period. The upregulated CXCR2 could facilitate cell proliferation, modulate the mRNA and protein expressions of type I collagen and matrix metalloproteinase 2 via JAK1/2‐STAT3 signalling pathway and inhibit cell migration via FAK/MLC_2_ pathway. The mechanism of the mechanotransduction may deserve further investigations, including the role such as microRNAs. microRNAs may play an important role in regulating cell proliferation, differentiation and apoptosis by binding to the 3^′^ untranslated region (UTR) of the target mRNA. Mechanical strain may induce significant changes in microRNAs of cells, suggesting that microRNAs might be one of the mechanisms that modulating ppHSFs under mechanical stimuli.^47^, ^48^ In conclusion, CXCR2 might be a potential therapeutic target for glaucoma from view of a sclera‐based therapy.

## AUTHOR CONTRIBUTIONS

**Chen Qiu:** Conceptualization (lead); data curation (lead); formal analysis (lead); investigation (lead); methodology (lead); visualization (lead); writing – original draft (lead); writing – review and editing (lead). **Chuandong Wang:** Data curation (lead); investigation (supporting); methodology (lead); visualization (lead); writing – review and editing (equal). **Xinghuai Sun:** Conceptualization (equal); formal analysis (equal); funding acquisition (lead); project administration (lead); supervision (lead). **Jianjiang Xu:** Methodology (lead); project administration (supporting); supervision (supporting). **Jihong Wu:** Formal analysis (supporting); methodology (lead); project administration (supporting). **Rong Zhang:** Methodology (equal); software (supporting). **Gang Li:** Methodology (equal); software (supporting). **Kang Xue:** Funding acquisition (supporting); methodology (supporting). **Xiaoling Zhang:** Conceptualization (lead); data curation (equal); funding acquisition (equal); methodology (lead); visualization (lead); writing – review and editing (lead). **Shaohong Qian:** Conceptualization (lead); data curation (lead); formal analysis (lead); funding acquisition (lead); supervision (lead); writing – review and editing (lead).

## FUNDING INFORMATION

This article was supported by grants from Natural Science Foundation of Shanghai (18ZR1406000), Shanghai Sailing Program (21YF1405400, 19YF1431200), National Key Research and Development Program of China (2020YFA0112700), State Key Program of National Natural Science Foundation of China (No. 82030027, No. 81430007), National Natural Science Foundation of China (No. 81830078, and No. 81802191, No. 81790641), top priority of clinical medicine centre of Shanghai (2017ZZ01020) and Shanghai Committee of Science and Technology (20Y11911200).

## CONFLICT OF INTEREST

The authors declared no conflict of interests.

## Supporting information


Figure S1

Figure S2

Figure S3

Figure S4

Figure S5

Figure S6

## Data Availability

The data that support the findings of this study are available from the corresponding author upon reasonable request.
