# Supplementary figures and images for: CXC‐ receptor 2 promotes extracellular matrix production and attenuates migration in peripapillary human scleral fibroblasts under mechanical strain
Source: J Cell Mol Med. 2022 Nov 8;26(23):5858–71. doi: 10.1111/jcmm.17609 (PMC9716229; doi:10.1111/jcmm.17609)

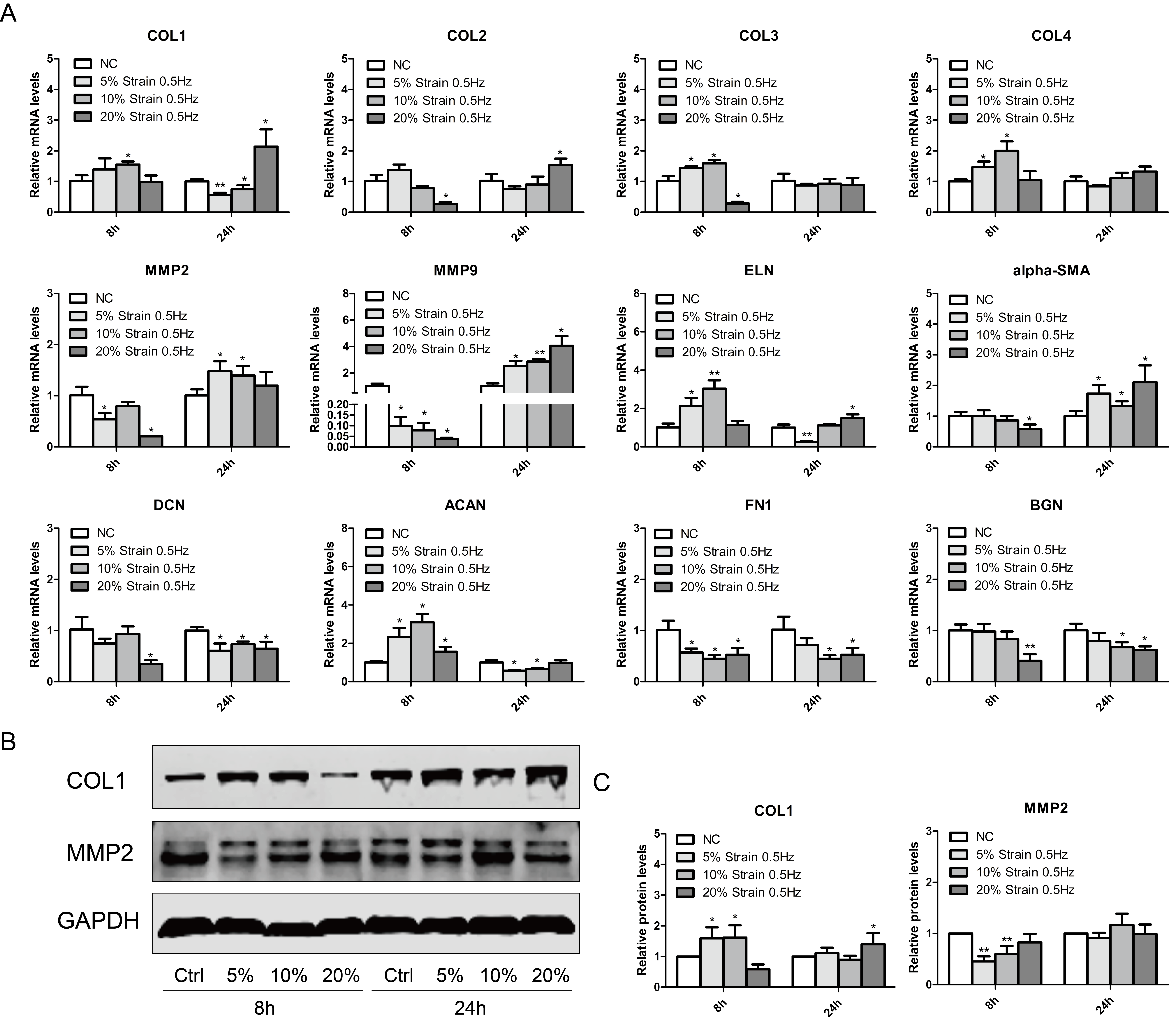

Supplement: Supplementary file 1 — Figure S1 [file JCMM-26-5858-s005.tif]

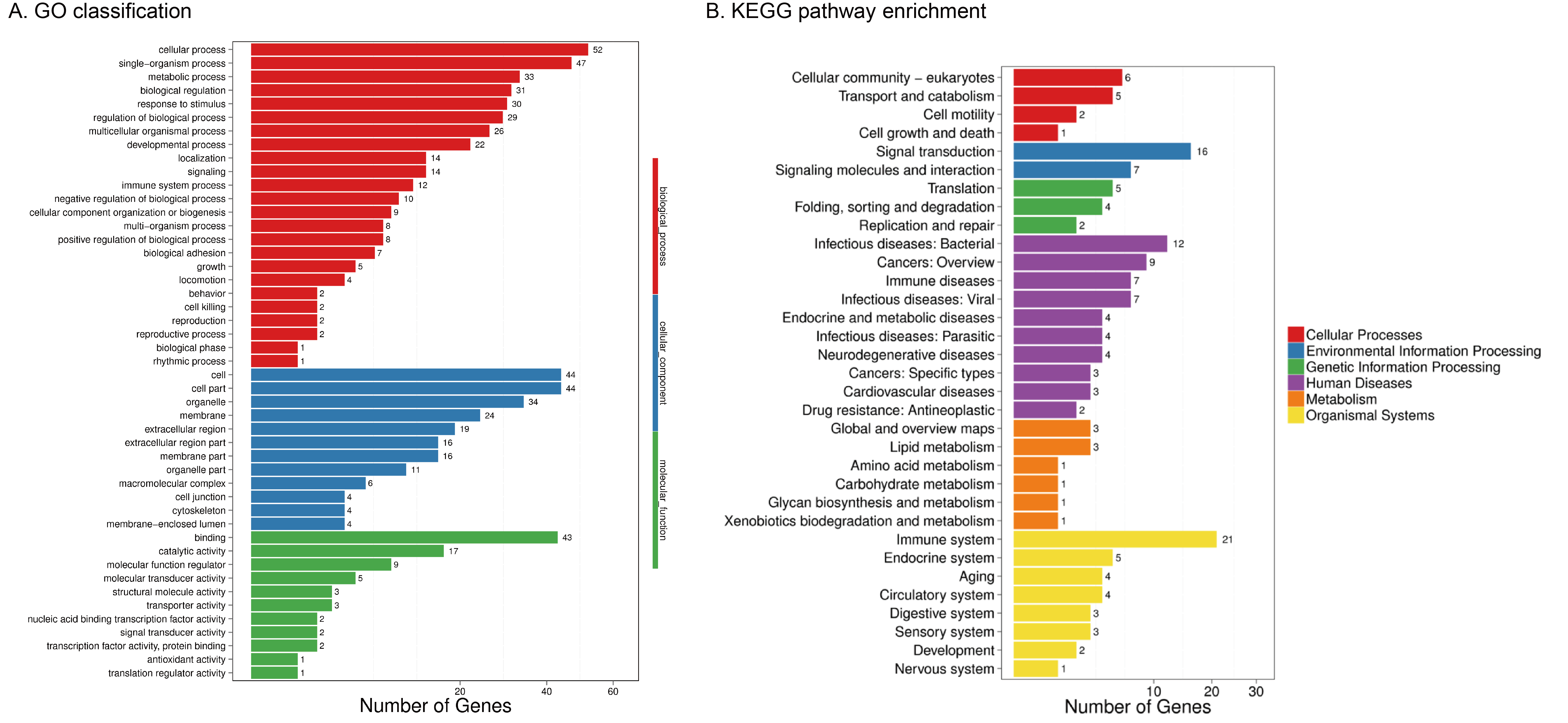

Supplement: Supplementary file 2 — Figure S2 [file JCMM-26-5858-s004.png]

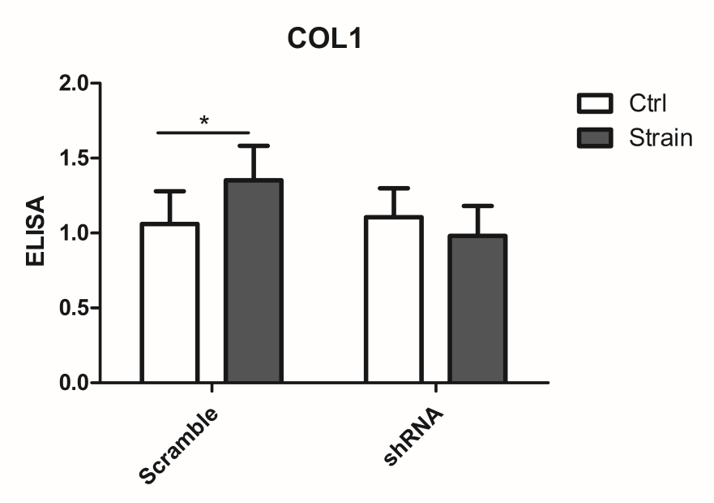

Supplement: Supplementary file 3 — Figure S3 [file JCMM-26-5858-s001.tif]

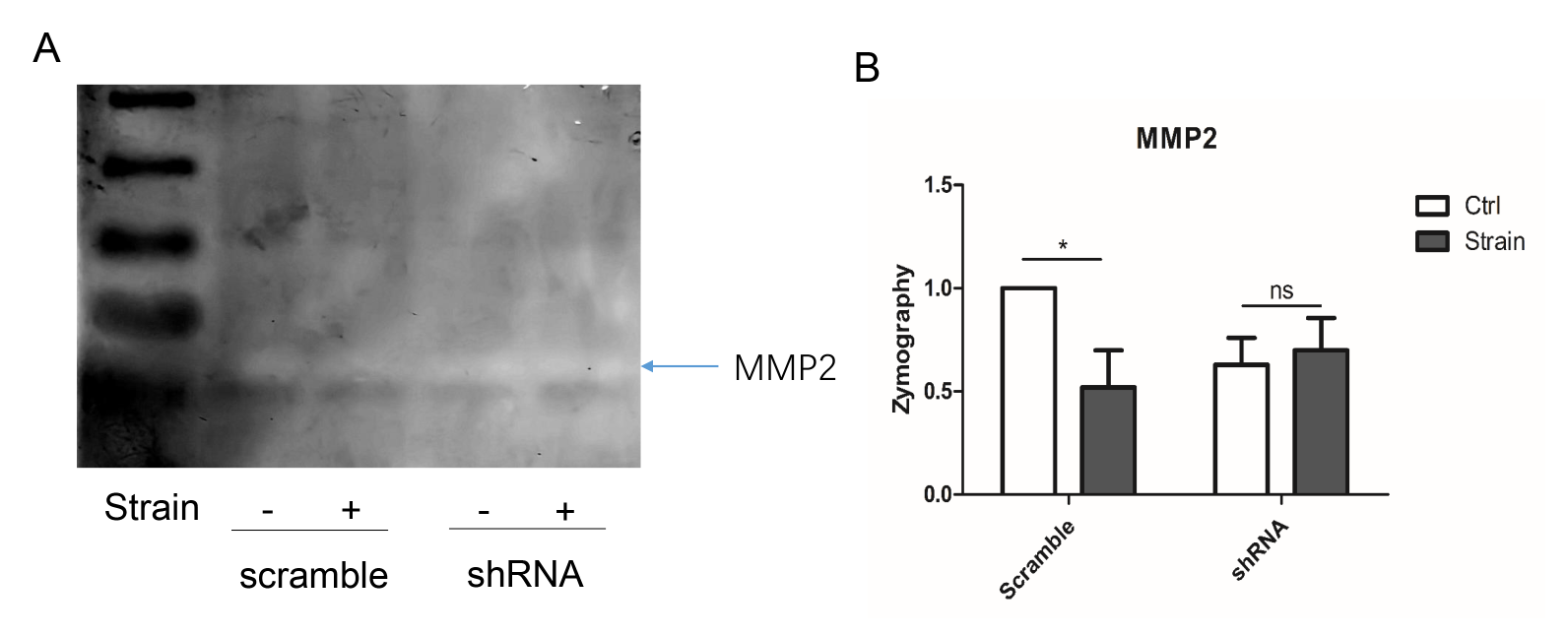

Supplement: Supplementary file 4 — Figure S4 [file JCMM-26-5858-s006.tif]

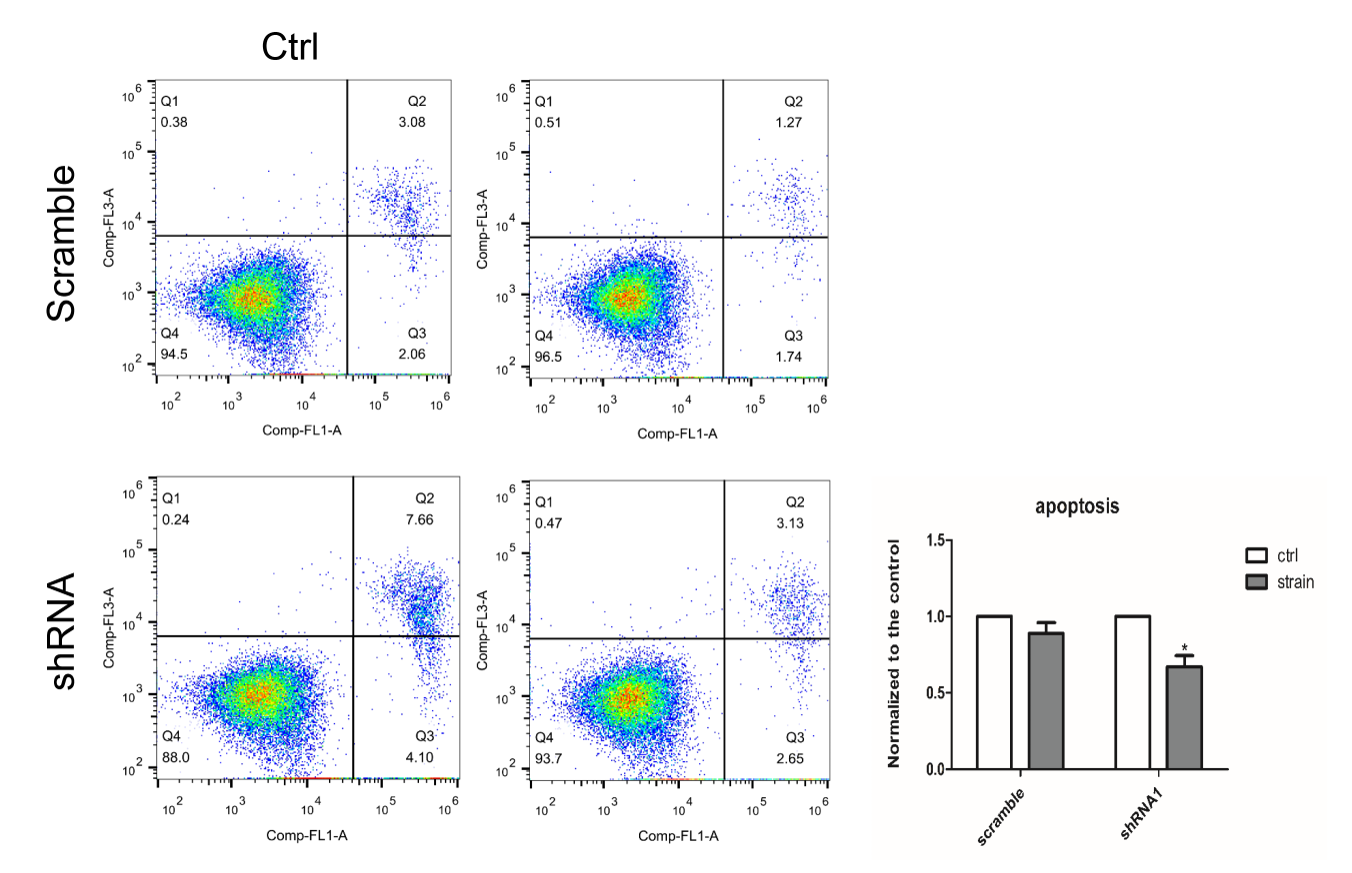

Supplement: Supplementary file 5 — Figure S5 [file JCMM-26-5858-s002.tif]

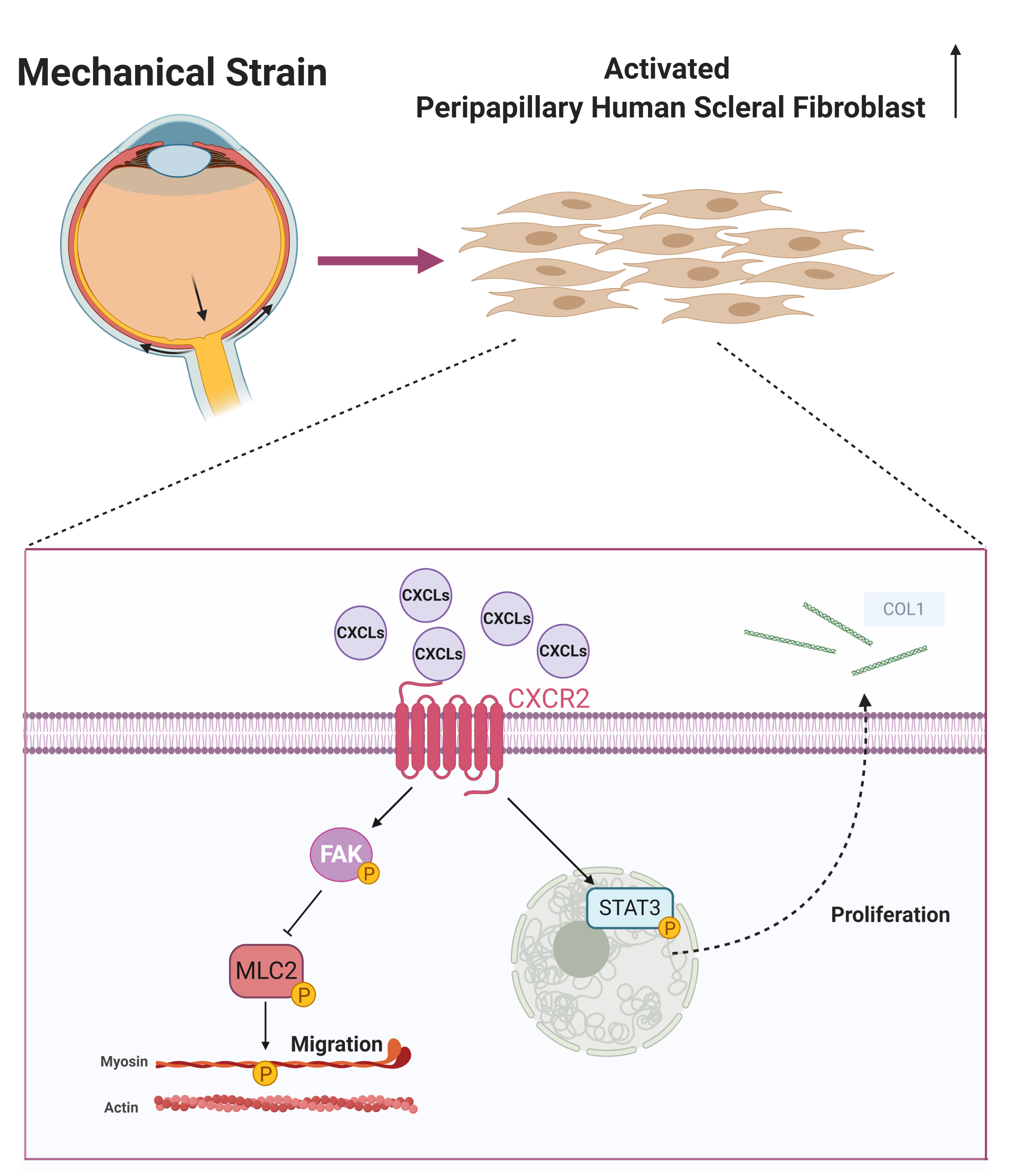

Supplement: Supplementary file 6 — Figure S6 [file JCMM-26-5858-s003.png]
